# Ready for Vaccination? COVID-19 Vaccination Willingness of Older People in Austria

**DOI:** 10.3389/fpubh.2022.859024

**Published:** 2022-06-03

**Authors:** Lukas Richter, Stephan Schreml, Theresa Heidinger

**Affiliations:** ^1^Department of Social Sciences, St. Pölten University of Applied Sciences, St. Pölten, Austria; ^2^Department of Socioeconomics, Vienna University of Economics and Business, Vienna, Austria; ^3^Department of Gerontology and Health Research, Karl Landsteiner University of Health Sciences, Krems an der Donau, Austria

**Keywords:** COVID-19, vaccination willingness, Austria, older people, vaccine hesitancy

## Abstract

In spite of findings highlighting higher health risk from infection compared to younger people, a certain percentage of older people in Austria still lack a valid vaccination certificate. The current gaps in vaccination coverage in countries such as Austria are likely to be in large part due to vaccination refusal and pose or will pose problems for the health system and consequently for all of society should the initial findings on Omicron coronavirus infectivity prove true. Surprisingly, only a few studies around the globe explicitly address older people's COVID-19 vaccination willingness. The present work therefore intends to contribute to this field by identifying factors associated with the decision for or against a vaccination among the older population in Austria. Data collected between late 2020 and early 2021 via the cross-national panel study Survey of Health, Aging and Retirement in Europe (SHARE) are used to perform multinomial logistic regression to analyse differences between COVID-19 vaccination supporters, undecided persons and rejectors. The results show that persons exhibiting a low risk assessment toward COVID-19, less health protection behaviors, lower education and belonging to households with financial burdens are significantly more likely to refuse vaccination or be ambivalent. Although multimorbidity reduces risk of vaccination refusal, poor subjective health was significantly related to a higher risk of refusing vaccination. The results point to the importance of addressing the factors related to refusal. Only by understanding these factors will it be possible to increase vaccination rates and thus minimize other restrictive measures.

## Introduction

Nearly 260 million confirmed infections and five million fatalities from or with COVID-19 (1), as well as estimations from the World Health Organization that another 700,000 people could die from COVID-19 by spring 2022 in the European region alone (2) are the grim results of the SARS-CoV-2 pandemic by the end of 2021. In Austria nearly 1.1 million infections and 12,000 deaths were recorded by November 2021 (3). At completion of this paper, the country has undergone multiple lockdowns, in order to relieve the health care system with the fourth nationwide lockdown starting at the end of November 2021 (4), even though the first COVID-19 vaccinations had already been administered on Dec. 27, 2020 (5) and have been freely available to all people in Austria for several months (6).

### Aim

Scientifically, vaccinations are indisputable as an effective means of combating infectious diseases (7) and, in the best case, have a reducing effect on infection, disease and transmission. Based on meta-analyses, COVID-19 vaccines have been shown to be highly effective in reducing infection and preventing severe disease progression (8, 9), additionally recent evidence suggests that transmission probability is reduced in vaccinated individuals (10). However, effective the vaccines may be, the desired consequence of this measures will only be achieved if there is an appropriate vaccination rate (11) and necessary amount of willingness in the population to get vaccinated. To achieve herd immunity, at least theoretically, recent estimates (based on data from Spain) suggest a good upper threshold of 70% for the ancestral variant, while the value for the delta variant is around 90% (12). However, it must be noted that these values vary according to multiple factors (12, 13), and that, even if the thresholds are reached, herd immunity does not constitute a panacea against the virus but requires adaptive and proactive measures.

Studies show that the COVID-19 vaccination acceptance rates at European (14) and at international level (15) varies significantly between countries. A recent review shows that of 114 countries, the acceptance rate is below 60% in 42 countries, whereby the situation is particularly diverse in Europe: At the beginning of 2021, the acceptance rate was 89% in Norway, 55% in Austria, and 47% in Hungary (16). As of December 7, 2021 (last check by the authors) 71.8% of the total population in Austria has had at least one COVID-19 vaccination and 67.6% has an active vaccination certificate (17). Even though the rapid increase of infections as of autumn 2021, followed by restrictions on unvaccinated persons and finally entering the fourth general lockdown as well as the multitude of public debates and subsequent announcement of a nationwide vaccination requirement starting in February 2022 (18) contributed to a slight increase in the coverage rate, which had been largely stagnant from August to November 2021 (17, 19), coverage rates continue to disappoint. It is noteworthy that even a certain percentage of older people in Austria continue to lack a valid vaccination certificate−18% among 55–64 year olds, 14% among 65–74 year olds, 10% among 75–84 year olds, and 13% among those 85 years and older (17)–in spite of findings highlighting higher health risk from infection for older persons as compared to younger people due to age-related physiological changes and multiple age-related comorbid conditions (20, 21). A large number of studies have demonstrated an age-related increase in health risk associated with COVID-19 infection (22, 23), which is reflected in more severe courses of disease and an increased risk of mortality (24, 25). This led many nations to prioritize their older population for vaccination at the advent of COVID-19 vaccines.

The current gaps in vaccination coverage in countries such as Austria are likely to be largely due to vaccination refusal despite availability of the vaccine (26), which poses health, economic, and ultimately social problems for society. The aim of this paper is to identify factors associated with the decision for or against COVID-19 vaccination among the older population using data of Austrian citizens.

### State of Research and Hypotheses

A growing body of scientific work on the topic of COVID-19 vaccination willingness can be identified which has already led to several literature reviews (27–31). For Austria, the authors are aware of four internationally published papers (32–35) to date. Surprisingly, only a few studies worldwide explicitly address older people (36–44). Current data availability is likely to play a role here, as the second SHARE Corona Survey data—which includes data on vaccination willingness—for example, are not yet available for scientific analysis, although initial results have already been published (36). Therefore, the present work is intended to expand the knowledge on this important group, which we currently know little about.

Rather than presenting a rundown of the full state of research, the most important results, as per the authors, are highlighted, which are used to formulate hypotheses and will then be empirically tested. Results of studies on COVID-19 vaccination willingness among older adults and literature reviews will be addressed. Studies dealing with the vaccination willingness of specific groups such as parents in relation to their children (45), adolescents (32) or healthcare workers (46) are excluded.

Important factors for the willingness to receive a COVID-19 vaccination were identified as the individual *risk assessment* of a COVID-19 infection or illness on one hand (28, 29, 37–39) and *past health protection behaviors*, such as having gotten an influenza vaccination in the past, on the other hand (28, 29, 37, 40). On a theoretical level, this can be explained by Protection Motivation Theory (47). Thus, willingness to be vaccinated becomes less likely when an infection is perceived as unlikely to occur or as posing a negligible threat to health or if the recommended protective action (in this case vaccination), is perceived as ineffective or even harmful in its own right (48). With regard to *health status*, it has been shown that pre-existing illnesses or a poor subjective health status are associated with a lower rate of vaccination refusal (29, 36, 37, 49). Perceived vulnerability associated with health status (43) and the anticipated risk of infection to health (39) can be used as arguments in this context. Among the socioeconomic factors, *financially disadvantaged* individuals were shown to be more likely to refuse vaccination (28, 29, 36, 42). *Higher education level* (28, 29, 31, 37, 40, 42) and *older age* (27–29, 31, 36, 40–42) have been found to be positively associated with vaccination acceptance. In addition, differences between genders surfaced in multiple studies: men were less likely to refuse vaccination as compared to women (27, 28, 31, 36, 37, 41, 42); however, a recent Italian study came to the opposite conclusion for older people (40). Despite these individual findings, meta-analyses also reveal divergent impacts of socioeconomic factors (27–30) depending, among other things, on the surveyed groups, sociocultural factors and different measurement methods. This emphasizes the importance of further study on the issue, especially among older people. Based on the presented state of research the following hypotheses can be formulated:

- H_1_: The lower the risk assessment, the higher the risk of refusing vaccination.
- H_2_: The less health protection behavior is shown, the higher the risk of refusing vaccination.
- H_3_: The better the health status, the higher the risk of refusing vaccination.
- H_4_: The lower the level of education and the worse the financial situation, the higher the risk of refusing vaccination.

Furthermore, a relationship between the degree of autonomy and the willingness be vaccinated is suspected, as it had been shown that perceiving vaccination as a social norm in a persons' circle of friends and family positively influenced the acceptance of vaccination (50). Under such conditions, it can be assumed that a high degree of individual autonomy is required (51) to refuse vaccination.

H_5_: The higher the level of perceived autonomy, the higher the risk of refusing vaccination.

## Methods

To test the hypotheses, data from the cross-national panel study Survey of Health, Aging and Retirement in Europe (SHARE) are used which includes data on persons aged 50 years and older living in the general population: Wave 8—COVID-19 Survey 1—Special Survey Austria (W8C19SAT) Release version: 1.0.0 (52) conducted from November 2020 to January 2021 in Austria via CATI serves were used as the main data source. In addition, data were imported from the main wave 8 survey (MW8) as well as from previous waves to minimize missing values (W7-W1). Table 1 shows the variables, their origin and distribution, whereby the values surveyed at the closest timepoint to the observation period (mainly W8C19SAT) were used for analyses.

**Table 1:** Operationalization.

**Variable**		**Manifestation**	**Distribution**	**Share Dataset**
Vaccination willingness		0 “supporter”	0 = 55%	W8C19SAT
		1 “undecided”	1 = 22,5%	
		2 “rejector”	2 = 22,5%	
Individual risk assessment	Risk of catching COVID-19	1 “very/low risk”	1 = 66%	W8C19SAT
		2 “medium risk”	2 = 28 %	
		3 “very/high risk”	3 = 6%	
	Risk to one's own health	1 “not/a bit dangerous”	1 = 16%	W8C19SAT
		2 moderately dangerous”	2 = 30%	
		3 “quite/very dangerous”	3 = 54%	
Health risk reduction	Currently social contact reduction	0 “yes”	0 = 94%	W8C19SAT
		1 “no”	1 = 6%	
	Influenza vaccination in 2019	0 “yes”	0 = 26%	W8C19SAT
		1 “no”	1 = 74%	
Health condition	Subjective health	1 “excellent/very good”	1 = 32%	W8C19SAT; W8,
		2 “good”	2 = 43%	
		3 “fair/poor”	3 = 25%	
	Multimorbidity	0 “2+ chronic diseases”	0 = 49%	MW8, W7, W6, W5, W4
		1 “none/one chronic disease”	1 = 51%	
Autonomy	Autonomy 3-items scale	1 (low)−18 (high).	*M* = 10,59 *SD* = 2,08	W8C19SAT
Sociodemographic variables	Highest formal education	ISCED 97 classification from 0 (no formal education)−6 (high formal education)	*M* = 3,39 *SD* = 1,29	W8C19SAT, MW8, W7, W6, W5, W4 W2, W1
	Able to make ends meet	0 “fairly/easily”	0 = 90%	W8C19SAT, MW8
		1 “with great/some difficulties”	1 = 10%	
	Age	1 “<65”	1 = 19%	W8C19SAT
		2 “65–79”	2 = 57%	
		3 “80+”	3 = 24%	
	Gender	0 “female”	0 = 61%	W8C19SAT
		1 “male”	1 = 39%	

*ISCED 97, International Standard Classification of Education Version 1997; M, mean; SD, standard deviation*.

### Sample Description

A total sample of *n* = 2,522 respondents in Austria serves as basis for analyses. 61% of the sample are women, the average age of the respondents is *M* = 72, 67 years (*SD* = 8, 54 years); 33% live alone, 56% in a two-person, and 11% in a three- or more-person household. 20% of the respondents have low (ISCED 0-2), 51% moderate (ISCED 3-4) and 29% have high formal education (ISCED 5-6). The more highly educated group is overrepresented, overall, the distribution structure can be described as sufficient; see Table 1.

### Operationalization

The dependent variable is nominally coded and differentiates between respondents who, during the survey period, (0) could imagine getting vaccinated against COVID-19 should such a vaccine be made available to them (55% hereafter referred to as “supporters”), (1) were undecided (22.5% “undecided”), and (2) were planning to refuse vaccination (22.5% “rejectors”).

Independent variables are divided into five dimensions—individual risk assessment, health protection behaviors, health condition, autonomy, and sociodemographic variables. For individual risk assessment, respondents were asked how threatening an infection with the SARS-CoV-2 virus would be (threat of virus) and how high they would estimate the probability of infection with the virus to their own person (probability of infection). Both items were measured using a 5-point rating scale and were grouped into three levels for analysis (see Table 1). For the dimension of health protection behaviors, a question on whether social contact had been reduced and another assessing whether the respondent had partaken in a vaccination against influenza in 2019 were included, these were answered with 0 “yes” and 1 “no”. Health condition is measured by subjective health assessment and multimorbidity. Multimorbidity is based on a longer list of questions (e.g., diabetes or high blood sugar etc.) and is coded as 0 “2+ chronic diseases” and 1 “none/one chronic disease”. This is due to the consideration that in comparison to persons with multimorbidity (reference group), the chance of belonging to the group of rejectors should increase for persons with no or only one chronic disease. Autonomy is measured using the subscale of the Psychological Well-being Scale of Ryff & Keyes (51) which calculates an additive index from 1 to 18 (low to high autonomy). According to Ryff & Keyes (51) a person with high scores should be independent and resists social pressure.

In addition, sociodemographic variables are included in the analyses. Education is classed into the International Standard Classification of Education (ISCED) for international comparability in SHARE [for more information, see the release guide of wave 8, see (52)]. For analyses, the variable ISCED97−0 = no formal education to 6 = high educational attainment—is used. Since ISCED data were largely unavailable at the time of the analysis in wave 8, this information had to be imported from previous survey waves. Financial situation is depicted using a question on the extent of difficulty for a household to make ends meet in a month; the 4-point scale was summarized in 0 “fairly/easily” and 1 “with great/some difficulties”. Age is coded as 1 “<65”, 2 “65–79” and 3 “80+”, gender as 0 “female” 1 “male”.

### Statistical Analysis

Data analysis was conducted using IBM SPSS 27 and unweighted data was used for analysis. To test the hypothesized associations multinomial logistic regression (a method that generalizes logistic regression to multiclass problems) was performed to analyse all three groups (supporters, undecided and rejectors) together. For the statistical model, the category “supporter” was chosen as the reference category in the dependent variable; accordingly, the effects of the independent variables are to be considered in relation to the reference group. In order to check the goodness of fit, independent logical regression models were additionally calculated—among others, the values of the area under the receiver operating characteristic curve (ROC AUC) are shown below.

## Results

For statistical requirements linearity was assessed using the Box and Tidwell (53) procedure and all metric variables were found to follow linearity to the logit. Goodness of fit for both independent models—supporters vs. undecided [Nagelkerke's *R*^2^ = 0.132 Hosmer–Lemeshow test (8) = 2,703; *p* = 0.952 and ROC AUC = 0.696] and supporters vs. rejectors [Nagelkerke's *R*^2^ = 0.273 Hosmer–Lemeshow test (8) = 2.730; *p* = 0.950 and ROC AUC = 0.777] are deemed acceptable. Consequently, the multinomial logistic regression with 2116 respondents was calculated [*X*^2^(30) = 440.238, *p* = 0.001]. Pearson's chi-square test [*X*^2^(3380) = 3419.328, *p* = 0.314] and deviance chi-square [X^2^(3380) = 3062.774, *p* = 1.00] indicate a good fit and Nagelkerke's *R*^2^ = 0.218 is deemed acceptable.

Table 2 shows the results of the multinomial logistic regression. Factors significantly associated with being in the group of undecided persons were found to be classification of risk of catching SARS-CoV-2 virus as moderate (odds ratio or OR 1.765), not having participated in the influenza vaccination in 2019 (OR 3.473), not having any or only one chronic disease(s) (OR 1.362) and reporting financial difficulties (OR 2.308). Being in very good health (OR 0.665), having a higher level of education (OR 0.843) and belonging to the 65–79 age group (OR 0.686) led to a reduction of probability to identify as undecided and a higher likelihood to be classed as a supporter of vaccination.

**Table 2:** Multinomial logistic regression vaccination willingness (supporter vs. undecided and supporter vs. refuser).

		**Undecided**				**Refuser**			
**Dimension**	**Variable**	**Odds Ratio**	**95% CI**		***p***	**Odds Ratio**	**95% CI**		***p***
Individual risk assessment	Risk of catching Corona (ref. very/high risk)								
	Moderate risk	**1.765**	1.024	3.042	0.041	1.076	0.631	1.834	0.788
	Very/low risk	1.477	0.870	2.506	0.148	1.211	0.728	2.013	0.461
	Dangerous for your health (ref. quite/very dangerous)								
	Moderately dangerous	1.192	0.912	1.559	0.198	**1.664**	1.258	2.202	0.000
	Not/a bit dangerous	1.223	0.851	1.757	0.276	**2.591**	1.853	3.623	0.000
Health defense behavior	Currently social contact reduction (ref. yes)								
	No	1.153	0.677	1.965	0.600	**2.083**	1.318	3.293	0.002
	Influenza vaccination in 2019 (ref. Yes)								
	No	**3.473**	2.589	4.660	0.000	**9.459**	6.235	14.348	0.000
Autonomy	1 (low autonomy)−18 (high autonomy)	0.973	0.922	1.027	0.316	1.033	0.977	1.092	0.255
Health condition	Subjective health (ref. fair/poor)								
	Good	1.025	0.759	1.384	0.872	0.798	0.585	1.089	0.155
	Very good/excellent	**0.665**	0.468	0.945	0.023	**0.525**	0.369	0.748	0.000
	Multimorbidity (ref. 2+ chronic diseases)								
	None/one chronic disease	**1.362**	1.067	1.739	0.013	**1.549**	1.205	1.991	0.001
Sociodemographic variables	ISCED 97 classification: 0 (no formal education)−6 (high formal education)	**0.843**	0.767	0.926	0.000	**0.826**	0.749	0.911	0.000
	Able to make ends meet (ref. fairly/easily)								
	With great/some difficulties	**2.308**	1.537	3.467	0.000	**3.267**	2.204	4.845	0.000
	Age (ref. 80+)								
	65–79	**0.686**	0.517	0.912	0.009	**0.724**	0.532	0.983	0.039
	<65	0.720	0.497	1.042	0.082	0.921	0.634	1.338	0.666
	Gender (ref. female)								
	Male	0.865	0.682	1.096	0.230	0.990	0.779	1.259	0.935
	X^2^/df/*p*	440.438/30/0.001							
	Nagelkerkes *R*^2^	0.218							
	*N*	2,116							
	Pearson's Chi^2^/deviance Chi^2^	3,419.328, *p* = 0.314/3,062.774, *p* = 1.00							

*Values marked in bold are significant*.

Persons were more likely to be classed as rejectors as compared to supporters if they assess the threat posed by COVID-19 as moderate (OR 1.664), or low (OR 2.591), did not currently reduce their social contacts (OR 2.083), did not get vaccinated against influenza in 2019 (OR 9.459), reported no or one chronic disease (OR 1.549) and had financial difficulties (OR 3.267). Like the comparison undecided/ supporters, a very good state of health (OR 0.525), higher level of education (OR 0.826) and belonging to the age group 65–79 (OR 0.724) lead to a reduction in likelihood to be part of the rejector group.

It is apparent, that the structure of *supporters vs. undecided* and *supporters vs. rejectors* is similar, with autonomy, health status and socioeconomic variables showing particularly striking similarity across both comparisons. Slight differences in individual risk assessment and health protection behavior can be observed: While undecided persons and supporters do not differentiate along the assessment of threat posed by COVID-19, the probability of being undecided increases when the own risk of infection is assessed as low. The reverse is true when comparing supporters and rejectors: while there is no differentiation along the assessment of own risk of infection, the probability of belonging to the group rejecting a vaccination increases when threat of a COVID-19 infection is assessed as lower. Correspondingly, as the threat of the virus is estimated as less severe, social contact reduction is seen less often in this group as compared to supporters. Results of the comparison of supporters vs. rejectors are discussed in more detail below.

## Discussion

Focussing on the first two dimensions (health risk assessment and health protection behaviors) it seems clear that a low perception of threat posed by the virus, lack of prior (influenza vaccination) and current (reduction of social contact) health protection behaviors increase the risk of being classed among the persons rejecting a COVID-19 vaccination. All of these factors thus indicate underestimation of risk and, according to the Protection Motivation Theory (47), make willingness to get vaccinated less probable. Therefore, H_1_ and H_2_ can be largely confirmed based on the data, with the notable limitation that supporters and rejectors do not differ in respect to the estimated risk of COVID-19 illness (probability of catching COVID-19). Based on Protection Motivation Theory it follows that it is not the perceived risk of getting infected with the virus, but rather the perceived consequences of such an infection for one's own health (severity of expected health problems), which fundamentally differentiates between these two groups. Conclusively, previous results are confirmed (29, 37).

The unexpected result concerning health status, which contradicts expectations set out in H_3_, must be considered as problematic: assessment of health status as good or fair reduces the risk of rejecting the vaccine by a factor of 0.53, i.e., a negative health assessment lead to a higher probability of refusing vaccination among older respondents. Not only do rejectors show a higher predisposition for infection due to their comparative lack of health protection behaviors but may also have an increased risk for severe courses of illness due to their poorer health status. Results using SHARE data from the summer of 2021 also showed an association between negative assessment of health status and more frequent refusal of vaccination in Bulgaria, Estonia, Latvia and Slovenia whereas no significant correlation has been found in other countries (36). However, as the second variable depicting health status (multimorbidity) followed the hypothesized direction (multimorbidity patients were less likely to reject the vaccination), H_3_ cannot be fully rejected.

H_5_ must be rejected based on the empirical analysis: Autonomy does not seem to be related to the decision to be vaccinated. At first glance, this may seem surprising, as the image of the “autonomous rejectors” is often perpetuated by the media. It is likely, that especially in the beginning of vaccination debates, both camps contained a broad cross-section of people with a high and low degree of autonomy. Further studies should examine this aspect, as the group of rejectors is or has become smaller probably due to increasing social pressure.

Hypothesis H_4_ can be confirmed by the present study and thus supports many of the international findings (28, 29, 36, 42). In short, the higher the level of education and the better the financial means, the lower the risk of refusing Covid-19 vaccination. In addition, gender has no influence, which is also confirmed by another study (36) for Austria, and the age group 65–79 years has a lower risk of refusing vaccination as compared to the oldest old (80+ years). Comparing our used data of the special SHARE survey Austria with the results of the second SHARE Corona Survey (36), a clear reduction of the group of “undecided” persons between the two survey time points—about 8 months apart—can be found, however the block of rejectors remained relatively strong in summer 2021 with 15% (in contrast to 22.5% in the end of 2020/early 2021).

As a limitation, it must be noted that the study presented only a fraction of variables, which is reflected in the level of Nagelkerke's R^2^. In particular, the exploration of the motivations for and against vaccination of the older population against the socioeconomic background could provide further insights. In view of the different acceptance rates in Europe, caution is required when generalizing the results also because the situation is currently undergoing rapid change. Further analysis is needed to better understand the remaining core of rejectors.

## Conclusion

This study points to the importance of understanding reasons for vaccination rejection. Only by understanding these factors it will be possible to increase vaccination rates and thus minimize other restrictive measures put in place to stop the pandemic spread. It seems particularly alarming that people with a poor subjectively health assessment had a higher risk of being among the refusers, and that socioeconomic status plays a considerable role. The question of how these groups can be activated for health measures, which has been raised before and will continue to be asked, will play an important part in the management, and hopefully the end of this health crisis. At least in Austria, the pandemic has proven once again that social inequalities become manifest in health behavior and, arguably, in health inequalities.

## Data Availability Statement

This paper uses data from SHARE Waves 1, 2, 4, 5, 6, 7 and 8 (DOIs: 10.6103/SHARE.w1.800, 10.6103/SHARE.w2.800, 10.6103/SHARE.w4.800, 10.6103/SHARE.w5.800, 10.6103/SHARE.w6.800, 10.6103/SHARE.w7.800, 10.6103/SHARE.w8.800, 10.6103/SHARE.w8ca.800), see Börsch-Supan et al. (2013) for methodological details.

## Author Contributions

LR was the primary author of this manuscript. Analysis and writing were done in collaboration with SS and TH. All authors contributed to the article and approved the submitted version.

## Funding

Under the terms of the Austria Open Access Publishing Framework Agreement, the St. Pölten University of Applied Sciences (Fachhochschule St. Pölten/FH St. Pölten) will cover Article Publishing Fees for eligible authors in any of the Frontiers journals. The SHARE data collection has been funded by the European Commission, DG RTD through FP5 (QLK6-CT-2001-00360), FP6 (SHARE-I3: RII-CT-2006-062193, COMPARE: CIT5-CT-2005-028857, and SHARELIFE: CIT4-CT-2006-028812), FP7 (SHARE-PREP: GA No. 211909, SHARE-LEAP: GA No. 227822, SHARE M4: GA No. 261982, and DASISH: GA No. 283646), and Horizon 2020 (SHARE-DEV3: GA No. 676536, SHARE-COHESION: GA No. 870628, SERISS: GA No. 654221, and SSHOC: GA No. 823782) and by the DG Employment, Social Affairs and Inclusion through VS 2015/0195, VS 2016/0135, VS 2018/0285, VS 2019/0332, and VS 2020/0313. Additional funding from the German Ministry of Education and Research, the Max Planck Society for the Advancement of Science, the United States National Institute on Aging (U01_AG09740-13S2, P01_AG005842, P01_AG08291, P30_AG12815, R21_AG025169, Y1-AG-4553-01, IAG_BSR06-11, OGHA_04–064, HHSN271201300071C, and RAG052527A) and from various national funding sources is gratefully acknowledged (see www.share-project.org).

## Conflict of Interest

The authors declare that the research was conducted in the absence of any commercial or financial relationships that could be construed as a potential conflict of interest.

## Publisher's Note

All claims expressed in this article are solely those of the authors and do not necessarily represent those of their affiliated organizations, or those of the publisher, the editors and the reviewers. Any product that may be evaluated in this article, or claim that may be made by its manufacturer, is not guaranteed or endorsed by the publisher.
